# Wearable-Derived Evening Motion Entropy and Morning Light Exposure Are Associated with Depressive Symptoms in University Students

**DOI:** 10.3390/bios16090513

**Published:** 2026-09-11

**Authors:** Guanxiang Ding, Qizhi Zhao, Linxin Zou, Yuezhou Zhang, Xianghong Zhao, Hao Li, Zhengxiang Yu

**Affiliations:** 1 School of Information Science and Engineering, NingboTech University, Ningbo 315100, China; 2 School of Information Science and Engineering, Zhejiang Sci-Tech University, Hangzhou 310018, China; 3 Department of Biostatistics & Health Informatics, Institute of Psychiatry, Psychology and Neuroscience, King’s College London, London WC2R 2LS, UK

**Keywords:** depressive symptoms, circadian rhythm disruption, light exposure, wearable sensing, machine learning

## Abstract

Depressive symptoms are prevalent in university students, yet scalable screening is constrained by self-report and intermittent assessment. Wearable monitoring of motion and light offers an objective alternative, though their combined screening characterization is limited. Undergraduate volunteers wore a self-designed wristband for seven days, recording triaxial acceleration and ambient light at 1 min resolution. Depressive symptoms were assessed via nine-item Patient Health Questionnaire (PHQ-9) and grouped as Dep (≥5) or HC (<5). Motion and light rhythm features were extracted from 24 h profiles and predefined windows; logistic regression (LR) and support vector machine (SVM) compared motion-only, light-only, and joint sets, interpreted with Shapley additive explanations (SHAP). After preprocessing, 152 participants provided valid activity data (HC = 132, Dep = 20) and 218 valid light data (HC = 186, Dep = 32). The Dep group showed delayed activity timing, lower daily light exposure, and greater evening motion irregularity, with differences most prominent during 18:00–22:00 for motion entropy and variance. The joint motion–light SVM performed best (accuracy 85.2%, recall 60.0%, F1 Score 46.2%, Area Under Curve 0.892). SHAP highlighted evening motion entropy and variance, morning light exposure, and rhythm-related wavelet features. These findings indicate that wearable motion and light rhythms are associated with depressive symptoms; evening motion entropy and morning light exposure may aid campus risk screening as interpretable markers, although the cross-sectional design and PHQ-9-based grouping preclude diagnostic or causal conclusions.

## 1. Introduction

Depressive symptoms are among the most common and burdensome mental health problems worldwide, contributing substantially to disability, suicide risk, and unmet care needs [1,2]. Among university students, subclinical depressive symptoms are particularly important because they may emerge during a period of academic pressure, social transition, and unstable daily routines. However, conventional approaches for assessing depressive symptoms rely largely on clinician-administered interviews and self-report questionnaires, which require active participation and are susceptible to recall bias and subjective reporting [3]. In addition, commonly used screening instruments are not sufficient for stand-alone diagnostic decision-making, especially when used outside specialist clinical settings [4]. These limitations highlight the need for scalable, objective, and low-burden approaches that can complement questionnaire-based screening in university populations.

Physical activity and environmental light exposure are closely linked to mental health and daily rhythm regulation. Prospective and longitudinal evidence suggests that lower physical activity and fewer daily steps are associated with greater depressive symptoms or higher risk of depression onset [5,6,7]. Light exposure is also relevant to mood because it contributes to circadian entrainment, sleep–wake timing, and rhythm stability. Large-scale observational evidence has linked outdoor light exposure with mood, sleep, and circadian-related outcomes [8], and clinical evidence indicates that appropriately timed light stimulation can alleviate depressive symptoms [9]. Nevertheless, many studies still rely on self-reported activity or outdoor exposure, which may introduce recall bias and limit the fine-grained characterization of daily behavioral rhythms.

Wearable devices provide a practical route for objective monitoring of physical activity, light exposure, and daily rhythmicity in real-world settings. Wearable and Internet-of-Medical-Things systems have been increasingly adopted to support mental disorder screening and prediction through passive physiological sensing [10]. Digital biomarkers derived from passive sensing have been increasingly used to characterize mood disorders and symptom changes [11]. Recent wearable and mobile-health studies have linked activity, sleep, seasonality, and multimodal sensing features with depression-related outcomes [12,13,14,15,16]. However, existing wearable studies often emphasize single modalities, commercial devices, or broad aggregate indicators such as step count, sleep duration, or total activity. Integrating multiple physiological-signal modalities through feature fusion has been shown to improve mental-health state classification [17]. Less attention has been given to low-cost wristband systems that jointly capture motion and ambient light exposure and translate these signals into clinically interpretable rhythm markers, such as activity timing, evening motion irregularity, and morning light exposure.

To address this gap, we developed a low-cost wristband to collect continuous motion and ambient light data from university students in a naturalistic campus setting. The present study aimed to examine whether wearable-derived motion and light rhythms are associated with PHQ-9-defined depressive symptoms and whether these features can support interpretable risk screening. Wearable health-monitoring systems commonly adopt machine learning, particularly support vector machines, to classify physiological signals directly from sensor measurements [18]. Specifically, we first characterized group differences in daily activity and light-exposure rhythms; second, we extracted time-window and rhythm-related features, with particular attention to evening motion entropy and morning light exposure; and third, we compared motion-only, light-only, and joint motion–light machine-learning models, using model-interpretability analysis to identify features that contributed most to depressive-symptom classification.

## 2. Materials and Methods

### 2.1. Study Design and Participants

**Figure 1:**
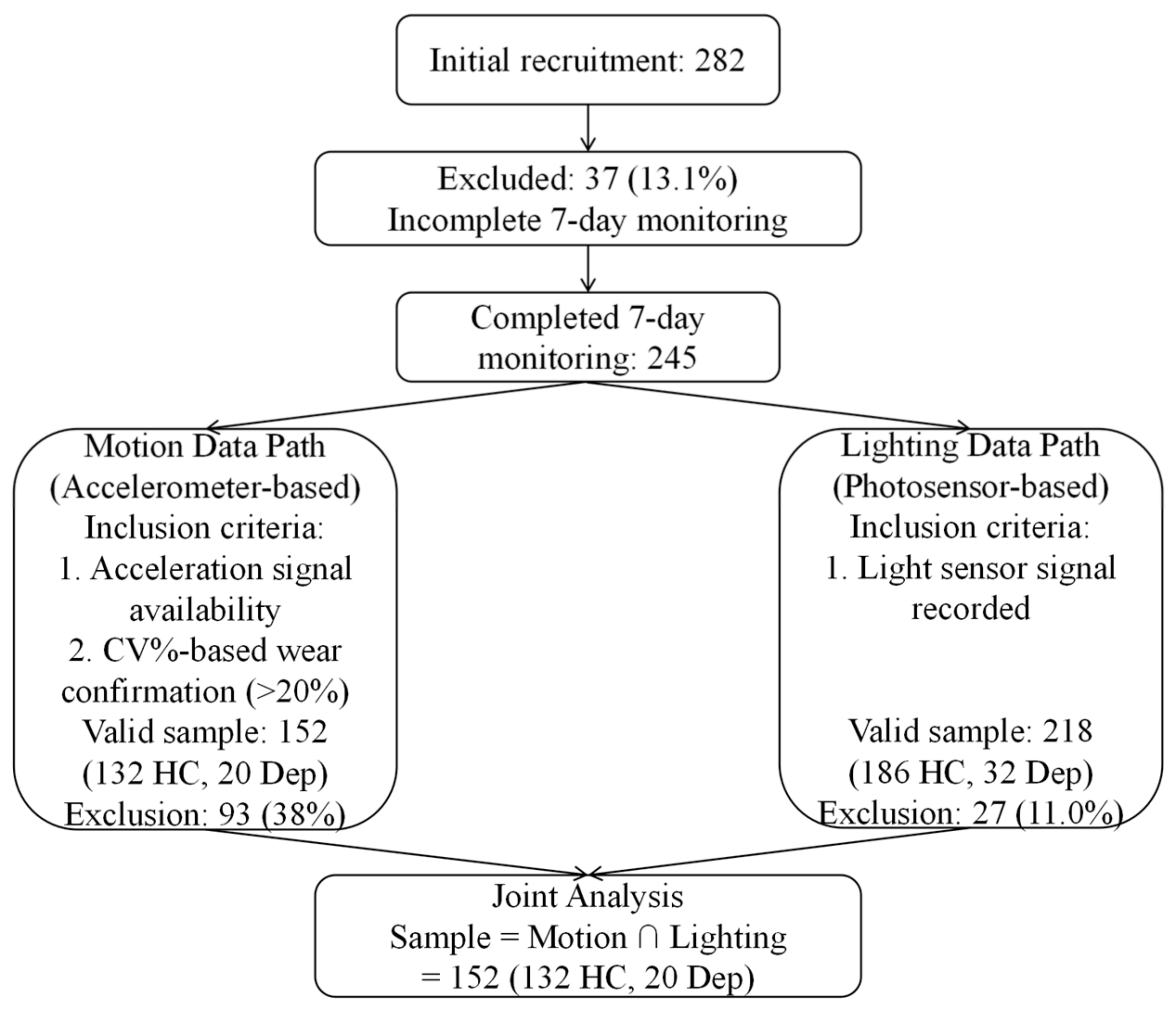
Participant screening and data selection flowchart for wristband-derived motion and light data. ∩ denotes the intersection of participants with both valid motion and valid light data.

The relevant data of this study were derived from a mental health research project conducted at NingboTech University (located in Ningbo City, Zhejiang Province, China). Participants were recruited from student volunteers at this university. Participant recruitment and wristband-based data collection were conducted from August 2025 to June 2026. All research activities involving human participants were performed after formal ethics approval was obtained on 8 August 2025. Participants completed the PHQ-9 before wearing the wristband and again after the 7-day wearing period; group assignment in the present analysis was based on the second PHQ-9 assessment obtained after wristband wearing. During the one-week wristband-wearing period, participants did not need to perform any additional operations. This study adopted a cross-sectional design. Ultimately, sensor data were screened using modality-specific criteria: lighting data were retained if the photosensor recorded any signal, whereas motion data required both acceleration signal presence and sufficient valid wear days determined by the coefficient of variation percentage (CV%)-based wear detection (daily mean CV% > 20%). PHQ-9 completeness was verified as part of the pre-specified quality-control criteria; all participants who completed the 7-day monitoring period provided complete PHQ-9 responses, and therefore no participant was excluded on this basis. The detailed participant screening and data selection process is illustrated in Figure 1.

### 2.2. Depressive Symptoms

The nine-item Patient Health Questionnaire (PHQ-9) comprises nine items, each rated on a four-point Likert scale (0 = not at all, 3 = nearly every day), yielding a total score of 0–27 [3,19]. In this study, the post-wearing PHQ-9 score was used for group assignment. Participants with a post-wearing PHQ-9 score ≥ 5 were assigned to the depressive-symptom group (Dep, encompassing mild-to-severe depressive symptoms), while those with a post-wearing PHQ-9 score < 5 were assigned to the healthy-control group (HC, indicating no depressive symptoms) [3,20].

This grouping follows the established clinical cutoff of 5, which is widely validated for identifying individuals with clinically relevant depressive symptoms [3,20,21]. Including mild depression cases is justified, as they not only impact quality of life but also carry progression risk, aligning with this study’s prevention-oriented focus on early detection.

### 2.3. Wristband Data Collection and Preprocessin

**Figure A1:**
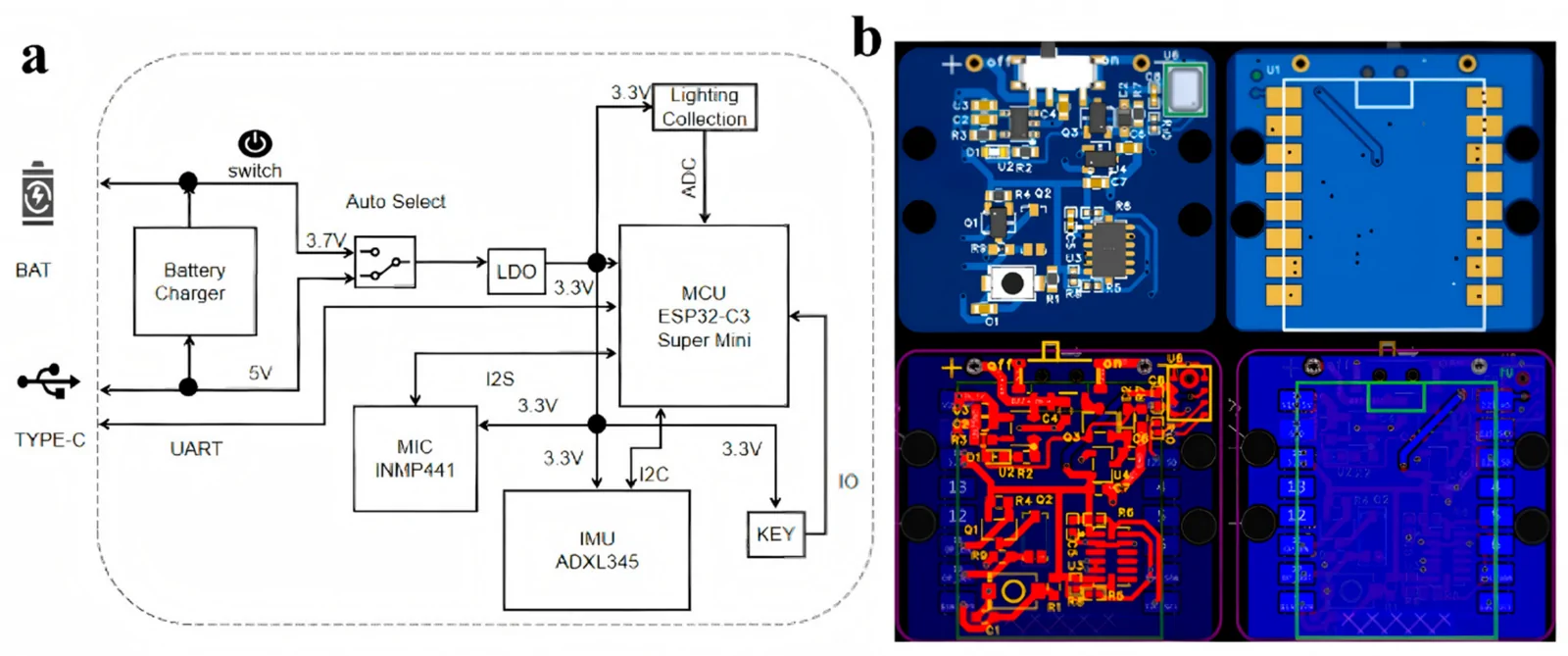
Block Diagram and Circuit Design of the Wristband: (**a**) Wristband Hardware Block Diagram; (**b**) Rendering of the Wristband PCB.

Participants wore a self-designed wristband that recorded timestamps, three-axis acceleration, and ambient light intensity at 1-min resolution. The device used an ESP32-C3 Super Mini microcontroller (Nologo, Shenzhen, China), an ADXL345 three-axis accelerometer (Analog Devices, Inc., Norwood, MA, USA), ±16 g full-resolution mode, 256 LSB/g), and a Vishay TEMT6000 × 01 ambient-light phototransistor (Vishay Intertechnology, Inc., Malvern, PA, USA). Hardware details are provided in Figure A1. To conserve battery, the firmware triggered a one-minute periodic acquisition (original sampling frequency: 1/60 Hz) via a timestamp-based real-time timer and stored the instantaneous sensor readings (the reported 1-min resolution is the native sampling interval, not a downsampled average). The composite acceleration parameter was computed as the signal vector magnitude (SVM):

(1) $$SVM\;=\;\sqrt{\left(x^{2}+y^{2}+z^{2}\right)}$$

where x, y, and z denote the instantaneous three-axis acceleration readings in LSB.

**Figure A2:**
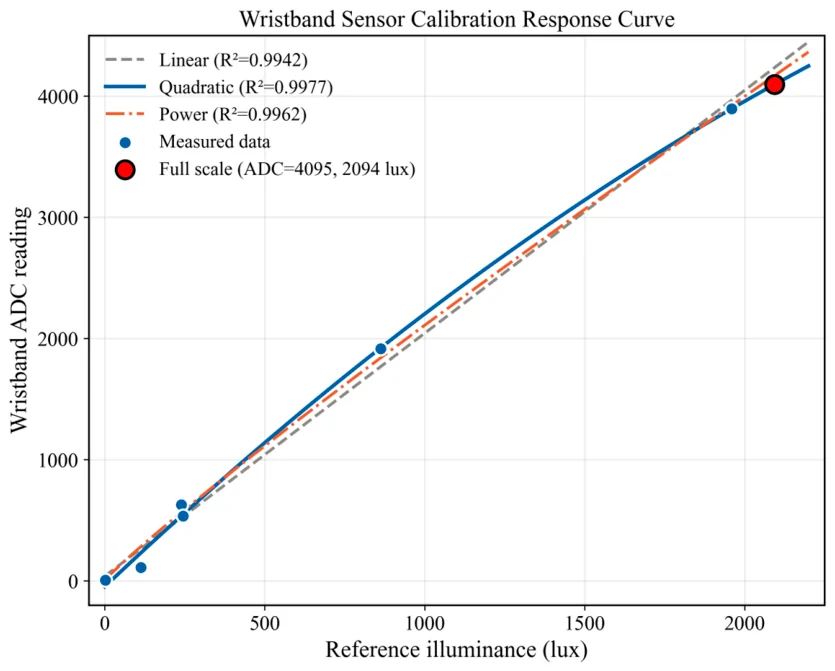
Photosensor response of the wristband.

Sensor performance was characterized in a dedicated validation experiment (Figure A2 and Table A1). Referenced against a non-standard lux meter (a smartphone ambient-light-sensor application (smartphone: iQOO 12, vivo Mobile Communication Co., Ltd., Dongguan, China), the analog-to-digital converter (ADC) output showed an excellent linear response over the valid range of 0–2000 lux (ADC = 2.00 × lux + 38.22; $R^{2}\;=\;0.9942$), with readings saturating at the 12-bit full scale above approximately 2000 lux. The accelerometer was configured with a ±16 g full-scale range and 13-bit resolution (256 LSB/g, 4 mg/LSB), with a static accuracy within 3.3% of the theoretical 1 g value, well within the sensor’s specified tolerance of ±5%.

**Figure 2:**
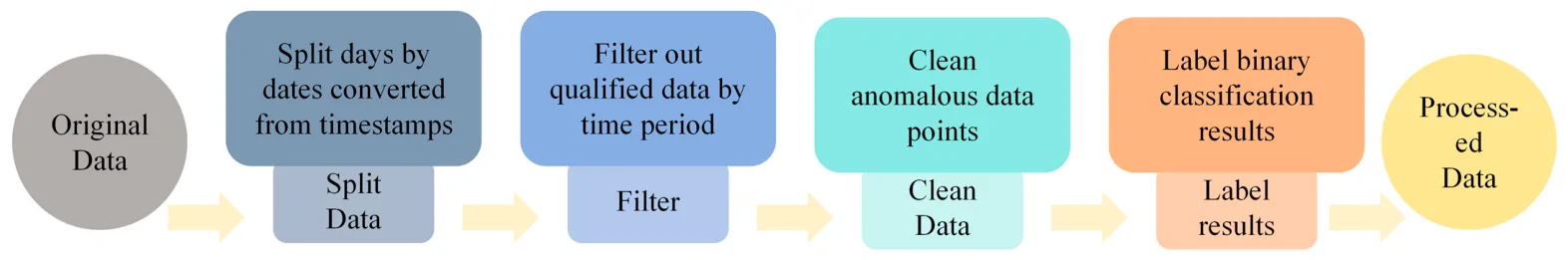
Data preprocessing workflow for wristband-derived motion and light data.

Raw data were processed using a four-step workflow: daily segmentation by timestamp, exclusion of incomplete first/last recording days and unqualified time periods, replacement of anomalous points using adjacent timestamps, and assignment of binary PHQ-9-based labels (Figure 2).

**Figure A3:**
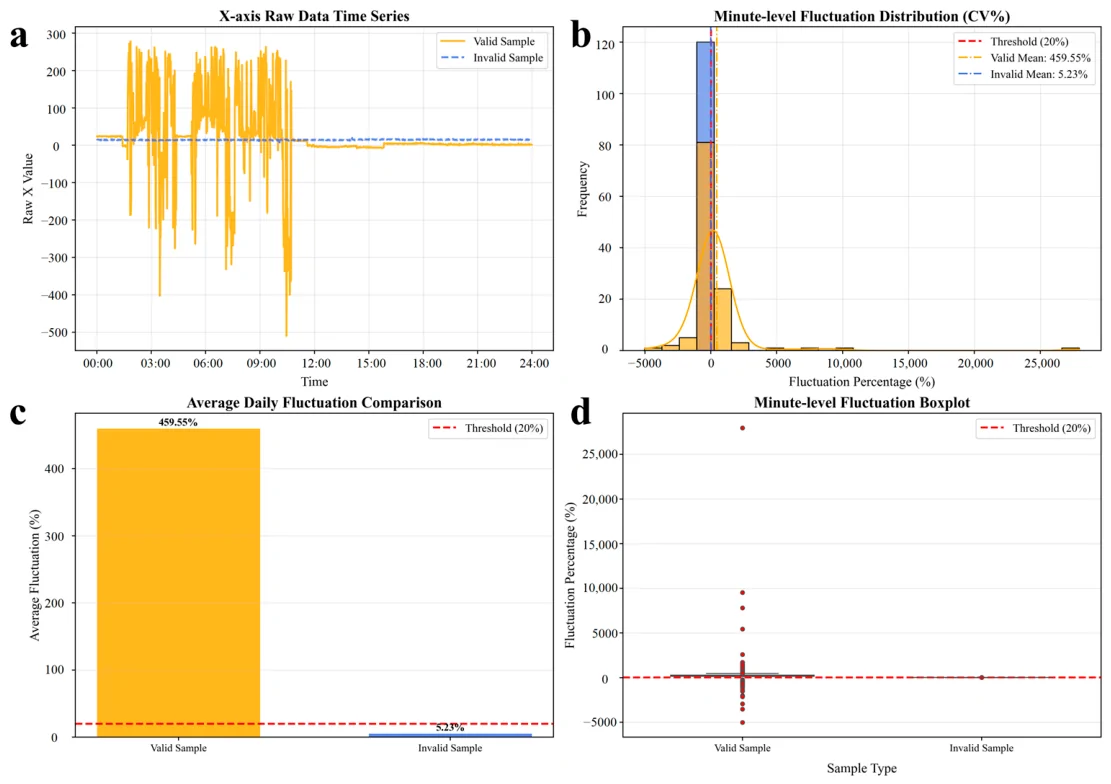
Comparison of acceleration signal characteristics between valid and invalid wear samples. (**a**) X-axis raw data time series. The orange solid line represents the valid sample, and the blue dashed line represents the invalid sample; (**b**) Minute-level fluctuation distribution (CV%). Histogram showing the frequency distribution of fluctuation percentage for valid (orange) and invalid (blue) samples. The red dashed line indicates the 20% CV threshold. The mean values are 459.55% for the valid sample and 5.23% for the invalid sample; (**c**) Average daily fluctuation comparison. Bar chart comparing the average fluctuation percentage between valid (orange) and invalid (blue) samples. The red dashed line indicates the 20% CV threshold. The values above the bars indicate the mean fluctuation percentages; (**d**) Minute-level fluctuation boxplot. Boxplots showing the distribution of fluctuation percentage for valid and invalid samples. The red dashed line indicates the 20% CV threshold. Individual dots represent outlier values beyond the whiskers.

Valid wear was assessed using the coefficient of variation (CV%) of consecutive 1-h-window *X*-axis acceleration after five-point median filtering. The CV% was calculated as:

(2) $$CV\%\;=\;\frac{SD}{mean}\;\times \;100\%$$

where SD and mean are the standard deviation and mean of the 60 X-axis samples within each 1-h window. The single-axis CV% thus isolates non-wear (device-stationary) periods without discarding genuine low-activity behavior, because even sedentary wear retains micro-postural gravity-projection drift that keeps window-level CV% above the cut-off. Daily average CV% was then computed as the mean of all window-level CV% values within that day. Days with daily average CV% > 20% were retained as valid wear, whereas days with CV% ≤ 20% were excluded as likely non-wear or stationary-device periods. This 20% cut-off was selected empirically: in this cohort, unworn or stationary-device days exhibited very low *X*-axis fluctuation (≈5.2% daily average CV%), whereas worn days showed substantially higher variability, and 20% cleanly separated the two regimes. The approach follows the established principle of variation-based non-wear detection [22], adapted here to a relative CV% metric that is robust to inter-unit gain and wearing-orientation differences. A comparison of CV% distributions between representative valid and invalid wear days is provided in Figure A3. The sensitivity of the 20% cut-off across different thresholds is assessed in Table A2, and the group-specific proportions of invalid/non-wear days in Table A3.

### 2.4. Time-Domain Analysis

For time-domain analysis, the three acceleration axes were combined into a minute-level composite acceleration. The daily mean composite acceleration was subtracted to emphasize time-varying motion, whereas light data were retained in their original scale to preserve exposure intensity.

Next, individuals were clustered by binary classification labels (HC vs. Dep groups), and average motion and light data were calculated separately for each group.

Finally, three data processing steps were implemented: filtering and smoothing via the application of a Savitzky–Golay filter to the data; curve fitting through high-order polynomial fitting conducted on both motion and light datasets; and light integration using Simpson’s numerical integration to calculate the area under the smoothed light curve (vs. the time axis) for quantifying total daily light exposure. Following these procedures, the time-domain datasets were finalized and used for subsequent data visualization.

### 2.5. Frequency-Domain Analysis

**Figure A4:**
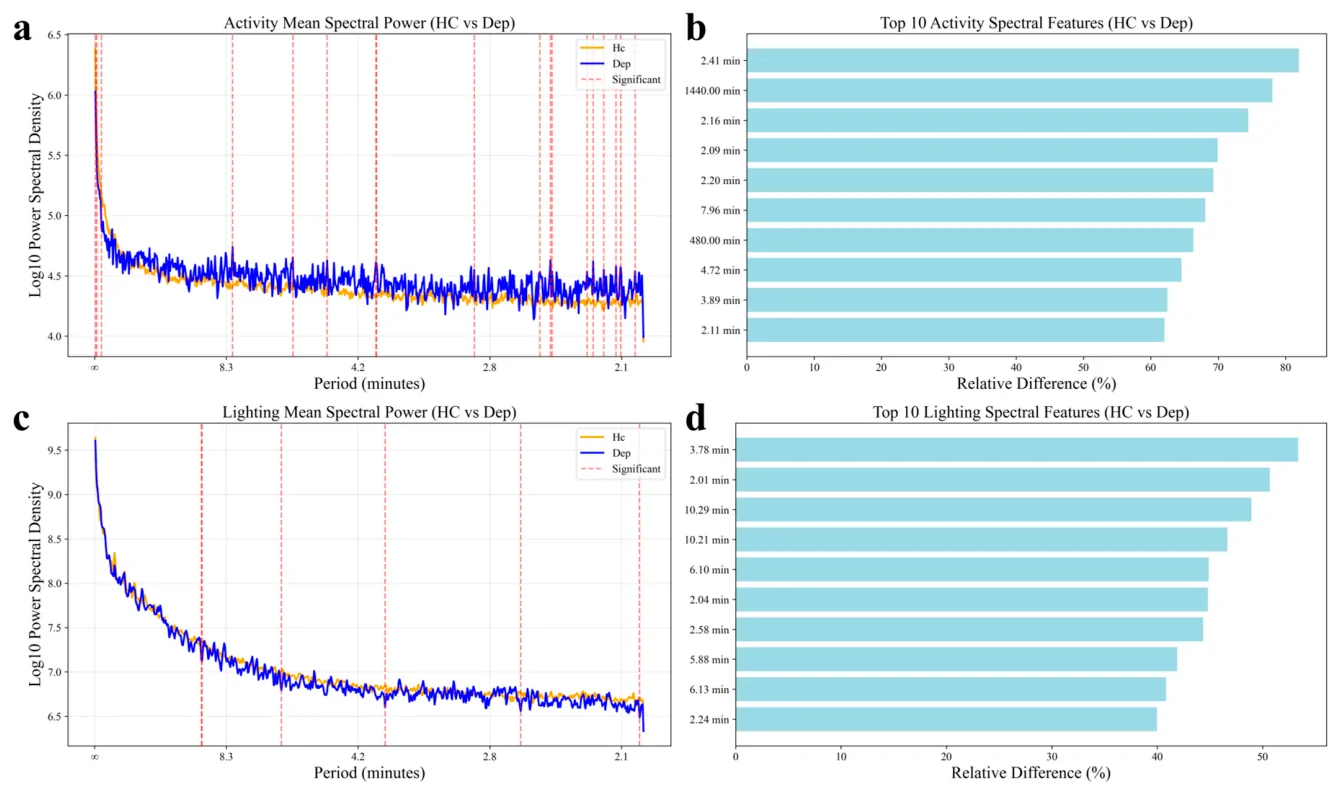
Comparison of Mean Spectral Power and Top 10 Spectral Feature Differences for Activity and Lighting: (**a**) Activity Mean Spectral Power Comparison Plot (HC vs. Dep); (**b**) Top 10 Activity Spectral Features Relative Difference Plot (HC vs. Dep); (**c**) Lighting Mean Spectral Power Comparison Plot (HC vs. Dep); (**d**) Top 10 Lighting Spectral Features Relative Difference Plot (HC vs. Dep). In panels (**a**,**c**), the orange and blue curves represent the HC and Dep groups, respectively, and the red dashed lines indicate significant frequency components. In panels (**b**,**d**), the bars represent the relative differences of the top 10 spectral features.

**Figure A5:**
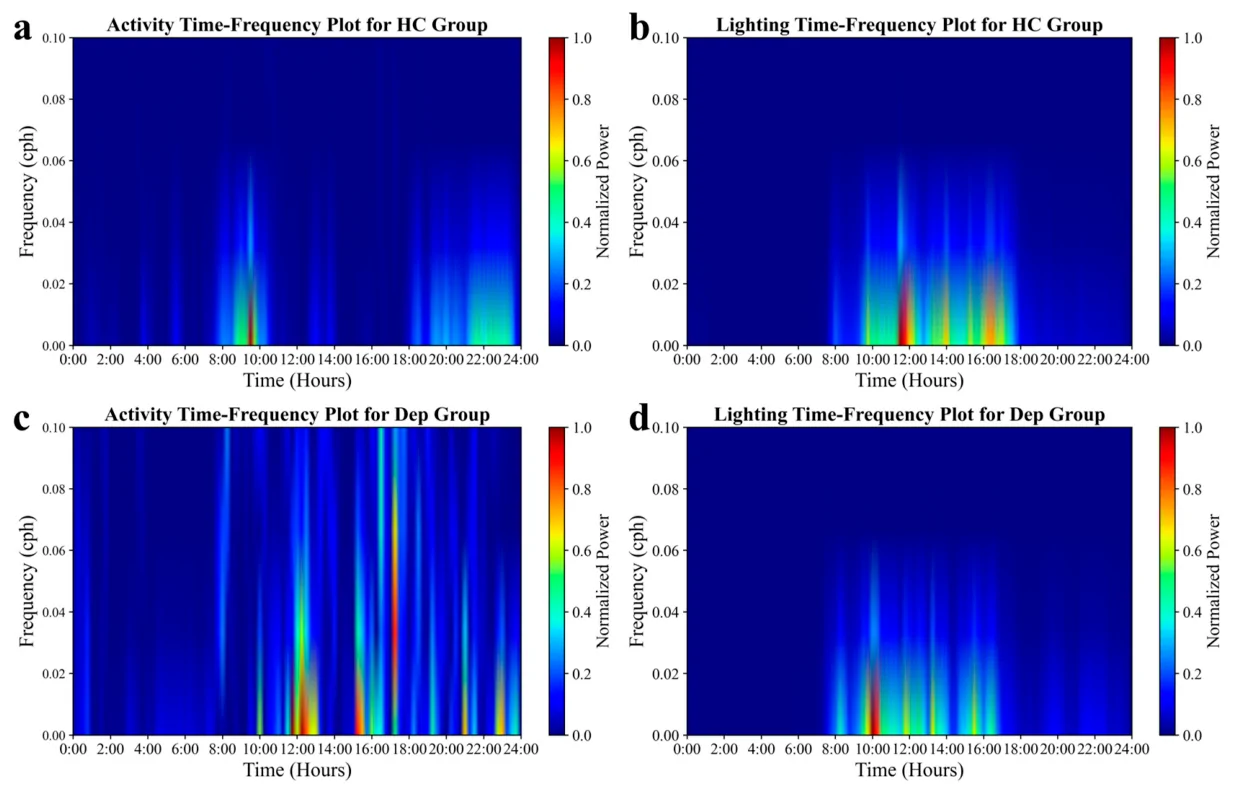
Time–Frequency Plots of Activity and Lighting for HC Group and Dep Group: (**a**) Activity Time–Frequency Plot for HC Group; (**b**) Lighting Time–Frequency Plot for HC Group; (**c**) Activity Time–Frequency Plot for Dep Group; (**d**) Lighting Time–Frequency Plot for Dep Group. In all panels, the horizontal axis represents time (hours), the vertical axis represents frequency (cycles per hour, cph), and the color scale indicates normalized power, ranging from low (blue) to high (red). Time–Frequency Plots of Activity and Lighting for HC Group and Dep Group: (**a**) Activity Time–Frequency Plot for HC Group, (**b**) Lighting Time–Frequency Plot for HC Group, (**c**) Activity Time–Frequency Plot for Dep Group, (**d**) Lighting Time–Frequency Plot for Dep Group.

Frequency-domain and time-frequency features were extracted as technical support for rhythm characterization and model construction. Detailed fast Fourier transform (FFT), short-time Fourier transform (STFT), and wavelet procedures, together with the corresponding spectral results, are provided in the Appendix A (Figure A4 and Figure A5, and Table A4).

### 2.6. Statistical Analysis

For statistical analysis, motion and light data were aggregated into five predefined daily time segments (0:00–6:00, 6:00–12:00, 12:00–18:00, 18:00–22:00, and 22:00–24:00). These windows were selected to reflect daytime activity, the evening transition period, and late-evening routines in student daily life. Six statistical features (mean, variance, median, skewness, kurtosis, and entropy via Gaussian kernel density estimation) were computed per participant, day, and segment. Kernel density plots visualized feature distributions, and Kolmogorov–Smirnov (KS) tests assessed inter-group distribution differences.

### 2.7. Machine Learning Modeling

Sixty-one features were extracted from each dataset via time-frequency domain kernel statistical analysis, forming three feature sets: motion, light, and their combined set. Motion-only and light-only models were trained using participants with valid data for the corresponding modality, whereas joint motion–light models used the intersection of participants with both valid motion and valid lighting data. For model training and evaluation, participants in each set were randomly divided into training and test subsets at an 8:2 ratio. These feature sets were separately input into logistic regression and support vector machine (SVM) models for training, prediction, and performance evaluation using four metrics: accuracy, recall, F1 score, and area under the receiver operating characteristic curve (AUC). Receiver operating characteristic (ROC) curves were generated for each model–feature set combination to visualize discriminative ability across thresholds, with AUC values derived directly from these curves.

To quantify feature contributions, Shapley additive explanations (SHAP) analysis—a game theory-based method using Shapley values—was applied to explore model interpretability. SHAP values decompose predictions into individual feature contributions relative to a baseline (e.g., mean sample prediction), with their sum equaling the difference between the model prediction and baseline. SHAP beeswarm plots were generated for visualization.

## 3. Results

### 3.1. Participant Characteristics

The valid participants included in this study after data preprocessing are summarized in Table 1, with information focused on three core dimensions: demographic attributes (gender and age) and binary classification results of the target condition.

### 3.2. Time-Domain Data Presentation

**Figure 3:**
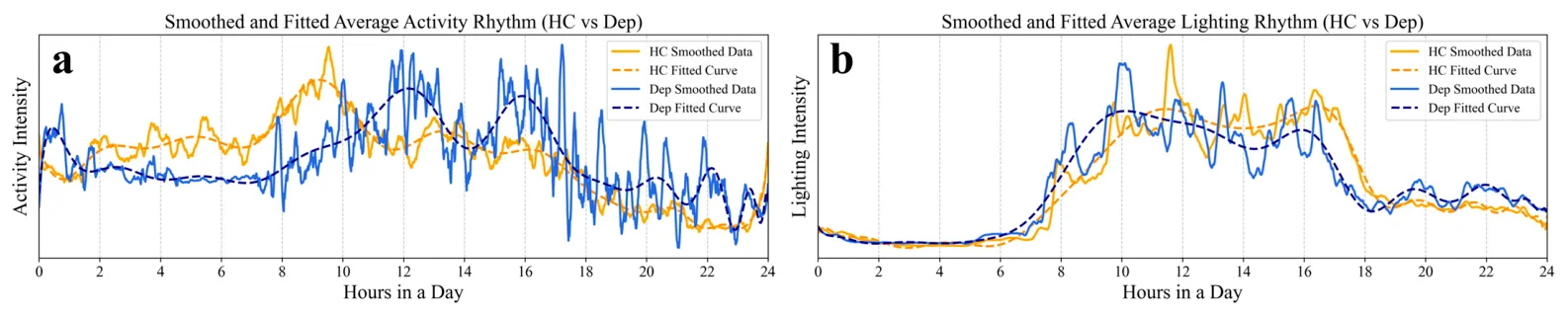
Filtering and fitting of mean-centered activity data and lighting data in the time domain: (**a**) mean-centered activity data; (**b**) lighting data.

Figure 3 shows the smoothed and fitted average activity and lighting rhythms of HC and Dep groups. Figure 3a presents the activity rhythms, with the horizontal axis representing hours in a day (0–24) and the vertical axis indicating activity intensity. The data here are the composite acceleration of movement for both groups after removing the DC component. Figure 3b displays the lighting rhythms, where the horizontal axis is hours in a day (0–24) and the vertical axis denotes lighting intensity, with the data being the original lighting intensity without DC component removal.

In the motion rhythm (Figure 3a), the HC group showed a main activity peak at approximately 09:00, whereas the Dep group showed a primary peak at approximately 12:00 and a secondary peak around 16:00. The Dep group also showed more fluctuation during nighttime hours. In the light rhythm (Figure 3b), both groups followed a broadly diurnal pattern, while cumulative daily light exposure was higher in the HC group than in the Dep group (mean difference: 300.32 digital units, based on raw photosensor readings, range 0–4095, intended for relative comparison only).

### 3.3. Statistical Presentation

Mean, variance, median, kurtosis, skewness, and entropy were calculated for the HC and Dep groups. A Kolmogorov–Smirnov (KS) test was conducted to assess group differences in the distributions of these statistical features. Kernel density plots for the six features were generated (Figure 3). Due to space limitations, only the kernel density plots of activity data from 18:00 to 22:00 are presented, where the horizontal axis represents feature values, the vertical axis denotes density, and dashed lines indicate the mean of each group’s statistical features (HC in orange, Dep in blue).

**Figure 4:**
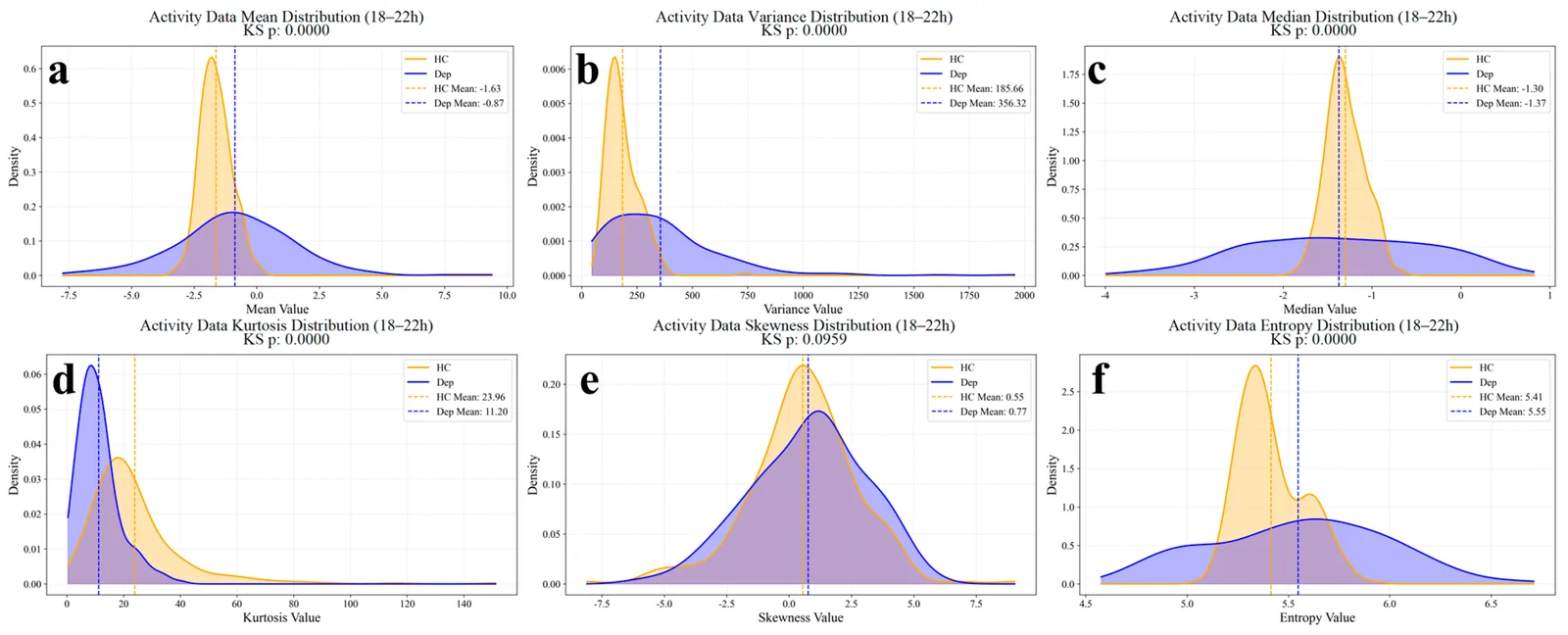
Distribution plots of statistical features of activity data from 18:00 to 22:00: (**a**) mean distribution plot of activity data; (**b**) variance distribution plot of activity data; (**c**) median distribution plot of activity data; (**d**) kurtosis distribution plot of activity data; (**e**) skewness distribution plot of activity data; (**f**) entropy distribution plot of activity data.

Figure 4 illustrates the kernel density estimation (KDE) plots for six statistical features derived from activity data during the critical 18:00–22:00 window. Statistical analysis using the Kolmogorov–Smirnov (KS) test revealed significant distributional disparities between the HC and Dep groups in terms of mean, variance, median, kurtosis, and entropy (all *p* < 0.001).

The Dep group showed wider distributions for several activity features during 18:00–22:00. Mean, variance, median, kurtosis, and entropy differed significantly between groups (all *p* < 0.001), whereas skewness did not reach statistical significance (*p* = 0.0959). Entropy and variance were higher in the Dep group than in the HC group.

### 3.4. Model Performance and SHAP Interpretation

**Figure 5:**
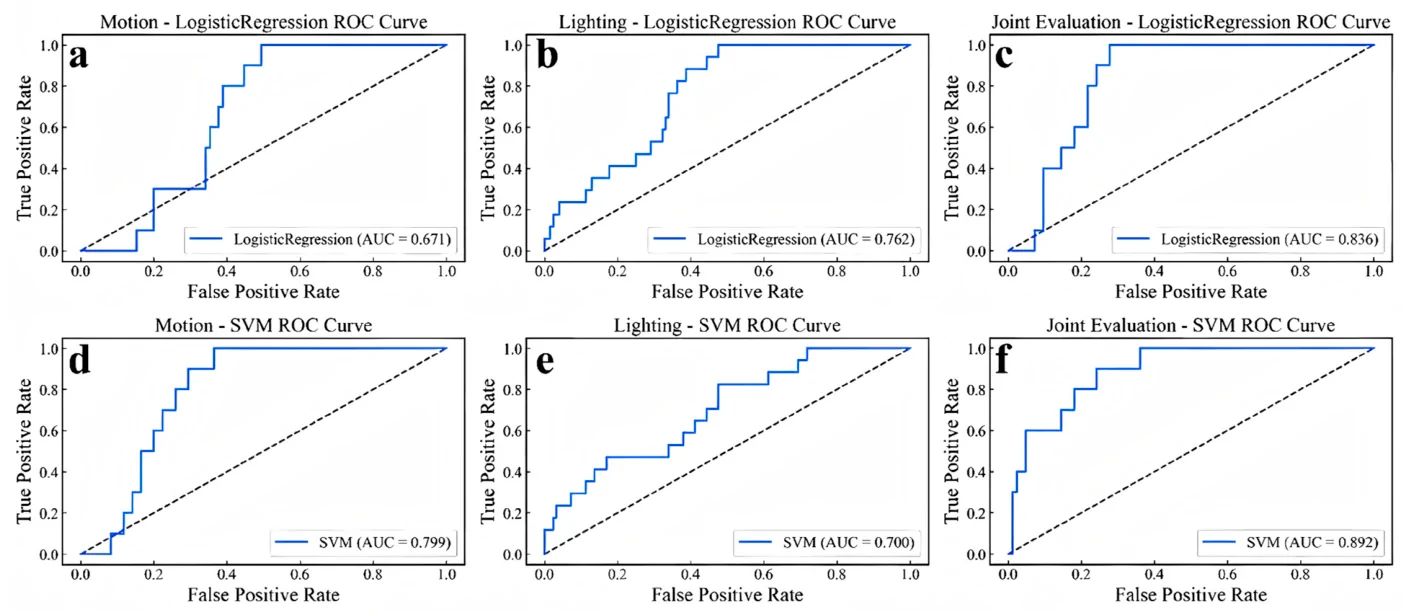
ROC curves of logistic regression and support vector machine models for activity and lighting data: (**a**) motion logistic regression (AUC = 0.671); (**b**) lighting logistic regression (AUC = 0.762); (**c**) joint motion–light logistic regression (AUC = 0.836); (**d**) motion SVM (AUC = 0.799); (**e**) lighting SVM (AUC = 0.700); (**f**) joint motion–light SVM (AUC = 0.892). The black dashed line represents the random classifier (AUC = 0.5).

Model performance and feature contribution were evaluated using ROC curves (Figure 5), classification metrics (Table 2), and SHAP plots (Figure 5). The joint feature set was constructed from the intersection of participants with valid motion and valid lighting data.

For logistic regression, the AUC increased from 0.671 with motion-only features and 0.762 with light-only features to 0.836 with joint features. For SVM, the AUC increased from 0.799 with motion-only features and 0.700 with light-only features to 0.892 with joint features.

The joint feature set also yielded the highest F1 scores in both models. In logistic regression, the F1 score increased from 28.6% with motion-only features and 28.1% with light-only features to 37.5% with joint features. In SVM, the F1 score increased from 38.1% with motion-only features and 26.2% with light-only features to 46.2% with joint features.

**Figure 6:**
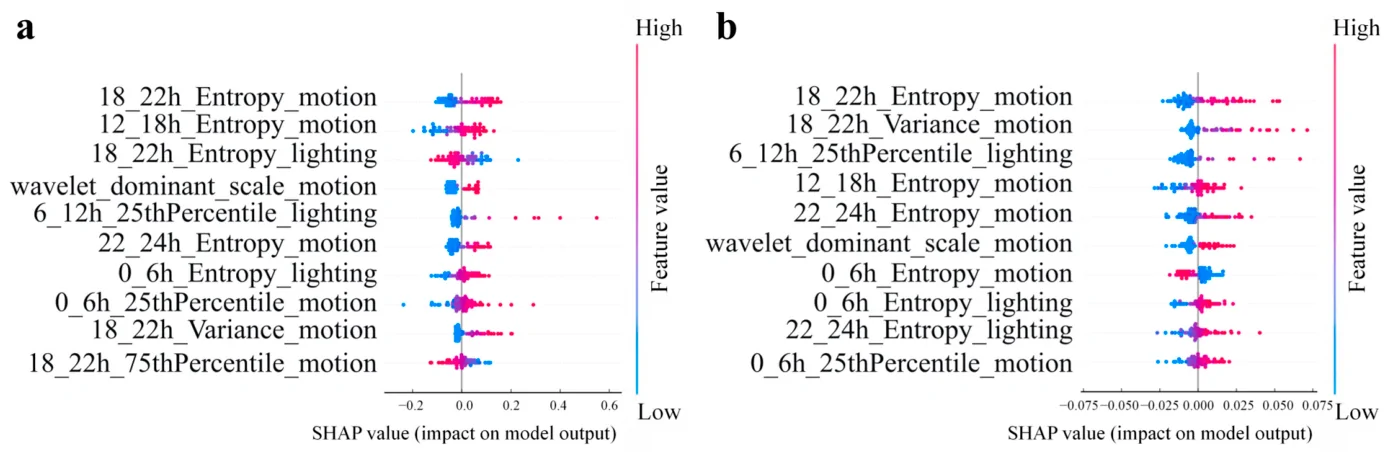
SHAP beeswarm plots of activity and lighting features under different models: (**a**) SHAP beeswarm plot of activity and lighting features under the logistic regression model; (**b**) SHAP beeswarm plot of activity and lighting features under the SVM model.

SHAP beeswarm plots (Figure 6a,b) summarized the contribution of the top 10 features to model predictions. Positive SHAP values indicate feature contributions toward the Dep group, whereas negative SHAP values indicate feature contributions toward the HC group. For each model, the top five features by mean absolute SHAP value are listed in Table 3 (logistic regression) and Table 4 (SVM), together with their physical interpretations and Dep/HC association directions.

Interpreting these directions in terms of the classification decision, for the logistic regression model the two motion-entropy features (18_22h and 12_18h) act as positive contributors: a participant exhibiting higher evening/afternoon motion irregularity receives larger positive SHAP values, which shift the linear combination toward the Dep side and raise the predicted depression probability. Conversely, the evening-light-entropy feature (18_22h_Entropy_lighting) exerts a negative contribution within the same model—higher evening-light irregularity yields negative SHAP values that push the prediction toward the HC side, so that a more regular (low-entropy) evening light profile is associated with a higher depression likelihood under logistic regression. This directional split means the LR classifier receives a Dep-indicative push from the two motion-entropy features that is partially offset by the HC-indicative (negative) SHAP contribution of the evening-light-entropy feature, so the two partially cancel each other out within the linear decision.

For the SVM model, all three top features are aligned in the same Dep-indicative direction: elevated evening motion entropy, elevated evening motion variance, and elevated morning-light lower bound each produce positive SHAP values that push the decision toward the Dep class. The absence of an internal direction conflict in the SVM top three suggests that, under the kernelized decision boundary, both motion and light abnormalities are encoded coherently as depression-associated signals.

Taken together, the shared leading feature 18_22h_Entropy_motion indicates that evening motion irregularity is the most consistent, model-agnostic discriminator: regardless of the classifier, higher evening motion entropy increases the predicted depression probability. Both models additionally placed a light-related feature among their top three, suggesting that the light modality also contributes to depression classification; however, the two light features are not the same descriptor (LR highlighted evening light entropy, whereas SVM highlighted the morning-light lower bound), and their SHAP directions partly diverged—LR’s evening light entropy was associated with the HC side, whereas SVM’s morning-light lower bound pointed toward the Dep side. This indicates that the light signal exerts a non-negligible but partly model-dependent influence on classification.

## 4. Discussion

This study used a low-cost wristband to collect 7-day motion and ambient-light data from university students and examined whether these wearable-derived rhythms were associated with PHQ-9-defined depressive symptoms. The main findings were that students in the Dep group showed delayed activity timing, lower cumulative daily light exposure, and greater evening motion irregularity; joint motion–light features outperformed single-modality feature sets; and model interpretation repeatedly highlighted evening motion entropy, evening motion variance, and morning light exposure. These findings suggest that depressive symptoms in students are reflected not only in the amount of daily activity or light exposure but also in the temporal organization and regularity of daily rhythms.

### 4.1. Rhythmic Instability as a Candidate Transdiagnostic Marker of Depressive Tendencies

The observed activity and light-exposure differences are consistent with the broader literature linking physical activity, light exposure, sleep–wake timing, and mood. Prospective and longitudinal evidence indicates that lower physical activity and fewer daily steps are associated with more severe depressive symptoms or increased depression risk [5,6,7]. In the present study, the Dep group did not merely show a simple reduction in activity; rather, its activity rhythm was delayed and more variable across the day. This pattern is relevant because rhythm instability may represent a trans-diagnostic regulatory feature across mental health problems [23], and young adults may be particularly sensitive to circadian preference and sleep-rhythm disruption [24].

### 4.2. Light Exposure and Multimodal Model Interpretation

Light exposure provides a second, complementary dimension for interpreting these findings. Outdoor and daytime light exposure have been associated with mood, sleep, and circadian-related outcomes in large population samples [8], and controlled light-based interventions can reduce depressive symptoms under appropriate circadian-stimulus conditions [9], In our data, the HC group showed higher cumulative daily light exposure than the Dep group. Although the present cross-sectional design cannot determine whether reduced light exposure contributes to depressive symptoms or reflects symptom-related behavioral changes, the pattern is consistent with a less consolidated day–night rhythm in students with depressive symptoms.

### 4.3. Model Interpretability and Screening Implications

The improvement of joint motion–light models over single-modality models suggests that activity and light exposure capture partially complementary information. This is consistent with digital-phenotyping frameworks in which passive sensing can capture real-world behavioral patterns that are difficult to obtain through retrospective questionnaires alone [11,14,15,16]. Importantly, the SHAP results indicated that the models were influenced by interpretable rhythm features rather than only by aggregate activity or light quantity. The prominence of evening motion entropy and variance suggests that depressive-symptom classification was sensitive to the stability of evening routines and rest–activity transitions, while the contribution of morning light exposure points to the potential role of daytime environmental entrainment. These markers are clinically readable and may be more actionable in student mental-health services than opaque high-dimensional features.

### 4.4. Practical Implications, Limitations, and Future Directions

From a practical perspective, a low-cost wristband-based approach may support non-invasive and passive risk screening in campus settings. Such a tool should not replace clinical interviews or validated questionnaires, but it could provide complementary information for identifying students who may benefit from follow-up assessment or supportive routine-based interventions. Similar wearable and mobile-health studies have shown the feasibility of using passive data streams for mood-related monitoring, while also highlighting challenges related to engagement, data completeness, and model generalizability [12,13,25,26].

Several limitations should be acknowledged. First, the sample was restricted to undergraduates from a single university, limiting generalizability. Second, the study was cross-sectional, so causal direction cannot be determined. Third, PHQ-9 was used as a screening instrument rather than a clinical diagnostic interview, and the PHQ-9 ≥ 5 threshold captures mild-to-severe depressive symptoms rather than diagnosed major depressive disorder. Fourth, the Dep group was smaller than the HC group, and the model lacked external validation. Fifth, the wristband was not subjected to controlled multi-unit calibration or standardized for wearing orientation and occlusion; however, because the light-exposure biomarkers are within-subject relative features, these residual variations do not bias the reported comparisons. Sixth, the observation window was limited to one week, which may not fully capture stable behavioral patterns and could be influenced by transient academic stressors such as exams or deadlines. Future studies with extended monitoring periods (e.g., 3–4 weeks) are needed to validate the observed trends and improve representativeness. Future multi-center and longitudinal studies should test whether evening motion entropy and morning light exposure predict subsequent symptom change and whether routine stabilization or increased daytime light exposure modifies these markers over time. On the hardware side, future deployments should incorporate controlled multi-unit calibration against a standard reference lux meter and a formalized wearing protocol (fixed sensor orientation, controlled occlusion) to further reduce unit-to-unit and wearing-factor variation; a wider-dynamic-range or saturation-aware sensing strategy should also be adopted given the 12-bit output clipping observed under direct outdoor sunlight.

## 5. Conclusions

This study evaluated whether continuously measured motion and ambient light rhythms from a low-cost wrist-worn device could provide interpretable markers of depressive symptoms in university students. Across seven days of naturalistic monitoring, students in the PHQ-9-defined Dep group showed a distinct organization of daily behavior and environmental exposure compared with the HC group. Their average activity rhythm was delayed, cumulative daily light exposure was lower, and motion during the 18:00–22:00 period was more irregular. In particular, evening motion entropy and variance differed markedly between groups, indicating that depressive symptoms may be reflected not only in the amount of daily activity or light exposure but also in the temporal organization and regularity of daily rhythms. These findings support the value of examining rhythm-related wearable features rather than relying solely on aggregate measures of activity or light exposure.

The machine-learning analyses further showed that motion and light exposure provided partially complementary information. Combining the two modalities improved discrimination compared with models using either modality alone. The joint motion–light SVM achieved the best overall performance, with an accuracy of 85.2%, recall of 60.0%, F1 score of 46.2%, and AUC of 0.892. Given the relatively small Dep group and the absence of external validation, these classification results should be interpreted cautiously. Nevertheless, the improved performance of the joint models suggests that motion and light exposure capture complementary aspects of depression-related daily rhythms. Importantly, SHAP analysis provided interpretable information about the features contributing to model predictions. Evening motion entropy was consistently among the most influential features across classifiers, while evening motion variance, afternoon and late-evening motion entropy, and light-related features, including morning light-exposure characteristics, also contributed to classification.

From a practical perspective, these findings suggest that low-cost wrist-worn sensing may support passive and low-burden risk screening in university settings. The most informative features identified in this study are clinically interpretable and potentially actionable. Irregular evening activity may reflect instability in evening routines and rest–activity transitions, while light-related features may capture differences in daytime environmental entrainment. Such wearable-derived information should complement, rather than replace, validated questionnaires or clinical assessments, and may help identify students who may benefit from follow-up assessment.

Several limitations should be considered when interpreting these findings. This was a cross-sectional study conducted at a single university, the Dep group was relatively small, and group assignment was based on the PHQ-9 rather than a structured clinical diagnosis. The models also lacked external validation, and the observed associations cannot establish causality or determine whether altered motion and light rhythms precede depressive symptoms or arise as consequences of them. Future studies should use larger, multi-center, and longitudinal cohorts, conduct external validation, and examine whether evening motion entropy and light-related rhythm features predict subsequent symptom changes. Overall, these findings indicate that the temporal organization of motion and light exposure may provide interpretable digital markers for depressive-symptom screening and offer a basis for future longitudinal wearable-based mental-health research.

## Figures and Tables

**Table 1:** Demographic information.

Data Set	Total	Male	Female	Age	HC	Dep
Activity	152	84	68	19 ± 0.5	132	20
Lighting	218	132	86	19 ± 1.0	186	32

**Table 2:** Classification performance metrics for different models and feature sets.

Model	Feature Set	Accuracy	Recall	F1 Score	AUC
Logistic regression	Activity	63.3%	70.0%	28.6%	0.671
	Lighting	67.2%	52.9%	28.1%	0.762
	Joint	**78.3%**	60.0%	37.5%	**0.836**
SVM	Activity	73.3%	80.0%	38.1%	0.799
	Lighting	68.1%	47.1%	26.2%	0.700
	Joint	**85.2%**	60.0%	46.2%	**0.892**

**Table 3:** Top 5 important features identified by the logistic regression model.

Feature Name	Physical Meaning	Correlation Tendency with Dep/HC
18_22h_Entropy_motion	Entropy of motion during the evening transition period (18–22 h), reflecting the irregularity of motion in the late-day window.	Higher values point toward Dep
12_18h_Entropy_motion	Entropy of motion during the afternoon period (12–18 h), reflecting the irregularity of motion across the afternoon.	Higher values point toward Dep
18_22h_Entropy_lighting	Entropy of light exposure during the evening transition period (18–22 h), reflecting the irregularity of evening ambient light.	Higher values point toward HC
wavelet_dominant_scale_motion	Dominant scale of the wavelet decomposition of the daily motion signal, indicating the temporal scale at which motion energy concentrates.	Higher values point toward Dep
6_12h_25thPercentile_lighting	25th percentile of morning light exposure (6–12 h), reflecting the lower range of morning ambient light intensity.	Higher values point toward Dep

**Table 4:** Top five important features identified by SHAP under the SVM model.

Feature Name	Physical Meaning	Correlation Tendency with Dep/HC
18_22h_Entropy_motion	Entropy of motion during the evening transition period (18–22 h), reflecting the irregularity of motion in the late-day window.	Higher values point toward Dep
18_22h_Variance_motion	Variance of motion during the evening transition period (18–22 h), reflecting the magnitude of evening motion fluctuation.	Higher values point toward Dep
6_12h_25thPercentile_lighting	25th percentile of morning light exposure (6–12 h), reflecting the lower range of morning ambient light intensity.	Higher values point toward Dep
12_18h_Entropy_motion	Entropy of motion during the afternoon period (12–18 h), reflecting the irregularity of motion across the afternoon.	Higher values point toward Dep
22_24h_Entropy_motion	Entropy of motion during the late-evening period (22–24 h), reflecting the irregularity of motion close to bedtime.	Higher values point toward Dep

## Data Availability

The data that support the findings of this study are available from the corresponding author upon reasonable request.

## Appendix A

**Table A1:** Ambient-light sensor response under representative indoor and outdoor illumination conditions.

Scenario	Reference Illuminance (Lux)	Wristband ADC	Status
Dark environment	0–5	0–8	Linear range
Indoor, curtains closed, lights on	109–113	75–198	Linear range
Indoor, curtains open, lights on	239–256	605–695	Linear range
Living room	245–268	510–542	Linear range
Desk lamp directly below	863–891	1836–1947	Linear range
Near desk lamp	1960–1976	3653–3896	Linear range
Outdoor, overcast	6072–6351	4095	12-bit saturation
Outdoor, direct sunlight	99,954–104,630	4095	12-bit saturation

Note: Reference values were obtained from a smartphone ambient light sensor application (non-standard lux meter). Both reference illuminance and wristband ADC values are presented as ranges of repeated simultaneous observations.

**Table A2:** Sensitivity of the daily–mean CV% non-wear cut-off across alternative thresholds.

Cut-Off	Day-Level Exclusion Rate (%)	Sample Retention (%)
10%	12.5	96.1
15%	16.5	94.7
20%	19.0	93.4
25%	21.3	92.1
30%	24.4	88.2

Note: Day-level exclusion rate is computed over recorded wristband days only and is not directly comparable to Table A3, whose denominator includes never-recorded analysis-window days.

**Table A3:** Group-specific invalid (non-wear/stationary device) day proportions by accelerometer (CV%-based).

Channel	Group	n (Participants)	Day-Level Exclusion Rate (%)
Motion (CV%)	HC	132	36.5
Motion (CV%)	Dep	20	48.0

Note: Day-level exclusion rate = 1 − [valid days ÷ (participants × 5-day window, excluding the incomplete device distribution and collection days)]. The denominator counts every day of the fixed 5-day window, including days with no usable recording (device not worn/data missing); the rate therefore combines CV-based non-wear failure with entirely missing days and is higher than the recorded-day CV-failure rate in Table A2 by design. The final joint analysis dataset is the intersection of motion-valid and light-valid days; this table reports the motion-channel CV%-based exclusion only, whereas the light channel applies a separate missing-recording criterion and is not subject to the CV% threshold.

**Table A4:** Test Results of the Top 5 Key Spectral Biomarkers.

Data Set	Rank	Period/min	Relative Differences Between Groups/%	*p* Value	Average Power of HC Group	Average Power of Dep Group
Activity.	1	2.41	81.98	0.000	17,827.48	42,592.63
	2	1440.00	78.07	0.013	2,432,490.93	1,066,643.32
	3	2.16	74.47	0.000	16,265.54	35,564.54
	4	2.09	69.92	0.000	18,057.03	37,470.68
	5	2.20	69.30	0.000	20,166.89	41,551.42
Lighting	1	3.78	53.38	0.019	6,905,464.10	3,996,048.12
	2	2.01	50.67	0.032	5,147,460.44	3,066,304.95
	3	10.29	48.92	0.009	22,557,750.82	13,690,623.69
	4	10.21	46.64	0.020	21,207,770.23	13,186,949.51
	5	6.10	44.89	0.063	11,367,945.75	7,200,445.25

Note: *p* values were calculated using a two-tailed independent samples *t*-test.

The period T in minutes was derived from the frequency f in Hz.

(A1) $$T=\frac{1}{f\times 60}$$

The relative difference between groups ${∆}_{rel}$ was calculated as the absolute difference between group means divided by their average:

(A2) $${∆}_{rel}\;=\;\frac{\left|\bar{{\mathrm{PSD}}_{\mathrm{HC}}}\;-\;\bar{{\mathrm{PSD}}_{\mathrm{Dep}}}\right|}{\frac{\bar{{\mathrm{PSD}}_{\mathrm{HC}}}\;+\;\bar{{\mathrm{PSD}}_{\mathrm{Dep}}}}{2}}\;\times \;100\%$$

where $\bar{{\mathrm{PSD}}_{\mathrm{HC}}}$ and $\bar{{\mathrm{PSD}}_{Dep}}$ are the mean power spectral densities of the HC group and the Dep group, respectively, at a given frequency.

The *t*-statistic was computed as follows:

(A3) $$t\;=\;\frac{\bar{{\mathrm{PSD}}_{\mathrm{HC}}}\;-\;\bar{{\mathrm{PSD}}_{\mathrm{Dep}}}}{\sqrt{\frac{S_{HC}^{2}}{M_{HC}}\;+\;\frac{S_{Dep}^{2}}{M_{Dep}}}}$$

where $S_{HC}^{2}$ and $S_{Dep}^{2}$ are the sample variances of the two groups at the given frequency, and $M_{HC}$ and $M_{Dep}$ are the sample sizes of the HC and Dep groups, respectively. The corresponding *p*-value is given by:

(A4) $$p\;=\;2\;\times \;P\;(T\;\geq \;\left|t\right|),\;T\;∼\;t(df),$$

With the degrees of freedom df approximated using the Welch–Satterthwaite equation:

(A5) $$df\;\approx \;\frac{{\left(\frac{S_{HC}^{2}}{M_{HC}}\;+\;\frac{S_{Dep}^{2}}{M_{Dep}}\right)}^{2}}{\frac{{\left(\frac{S_{HC}^{2}}{M_{HC}}\right)}^{2}}{M_{HC}\;-\;1}+\frac{{\left(\frac{S_{Dep}^{2}}{M_{Dep}}\right)}^{2}}{M_{Dep}\;-\;1}}$$

The mean power spectral density for each group was calculated as follows:

(A6) $$\bar{{PSD}_{HC}}\;=\;\frac{1}{M_{HC}}\;\sum_{i=1}^{M_{HC}}{PSD}_{HC,i}$$

(A7) $$\bar{{PSD}_{Dep}}=\frac{1}{M_{Dep}}\;\sum_{i=1}^{M_{Dep}}{PSD}_{Dep,i}$$

where ${PSD}_{HC,i}$ and ${PSD}_{Dep,i}$ are the power spectral density values of the i-th participant in the HC and Dep groups, respectively, at a given frequency. The power spectral density was estimated using the periodogram method:

(A8) $$PSD(f_{k})\;=\;\frac{1}{N\cdot f_{s}}{\left|\sum_{n=0}^{N-1}x_{n}e^{-j2\pi f_{k}n}\right|}^{2}$$

where $x_{n}$ is the time series of length N = 1440 (one measurement per minute over 24 h), $f_{s}\;=\;\frac{1}{60}$ Hz is the sampling frequency, and $f_{k}$ is the k-th frequency bin.
